# An Evaluation of Mobile Health Application Tools

**DOI:** 10.2196/mhealth.3088

**Published:** 2014-05-01

**Authors:** Preethi R Sama, Zubin J Eapen, Kevin P Weinfurt, Bimal R Shah, Kevin A Schulman

**Affiliations:** ^1^Duke Clinical Research InstituteDuke University School of MedicineDurham, NCUnited States; ^2^Department of MedicineDuke University School of MedicineDurham, NCUnited States; ^3^Department of Psychiatry and Behavioral SciencesDuke University School of MedicineDurham, NCUnited States

**Keywords:** cellular phone, Internet, medical informatics applications, social media

## Abstract

**Background:**

The rapid growth in the number of mobile health applications could have profound significance in the prevention of disease or in the treatment of patients with chronic disease such as diabetes.

**Objective:**

The objective of this study was to describe the characteristics of the most common mobile health care applications available in the Apple iTunes marketplace.

**Methods:**

We undertook a descriptive analysis of a sample of applications in the “health and wellness” category of the Apple iTunes Store. We characterized each application in terms of its health factor and primary method of user engagement. The main outcome measures of the analysis were price, health factors, and methods of user engagement.

**Results:**

Among the 400 applications that met the inclusion criteria, the mean price of the most frequently downloaded paid applications was US $2.24 (SD $1.30), and the mean price of the most currently available paid applications was US $2.27 (SD $1.60). Fitness/training applications were the most popular (43.5%, 174/400). The next two most common categories were health resource (15.0%, 60/400) and diet/caloric intake (14.3%, 57/400). Applications in the health resource category constituted 5.5% (22/400) of the applications reviewed. Self-monitoring was the most common primary user engagement method (74.8%, 299/400). A total of 20.8% (83/400) of the applications used two or more user engagement approaches, with self-monitoring and progress tracking being the most frequent.

**Conclusions:**

Most of the popular mobile health applications focus on fitness and self-monitoring. The approaches to user engagement utilized by these applications are limited and present an opportunity to improve the effectiveness of the technology.

## Introduction

### Mobile Devices and mHealth

The development of mobile communications devices such as smartphones and tablet computers has spurred rapid growth in the field of mobile health (mHealth), the use of mobile-enabled applications that collect or deliver health care information and data. These applications offer the potential for dynamic engagement of patients and providers in health care and a new means of improving health outcomes. This technology could have profound application in the prevention of cardiovascular disease or in the treatment of patients with chronic disease such as diabetes and congestive heart failure. The rapid growth in mHealth has outpaced the science needed to validate the clinical effectiveness (and safety) of health-related applications. Due to the proliferation of smartphones and health-centric mobile applications (app), the US Food and Drug Administration recently issued guidance that will apply a similar risk-based approach to assure the safety and effectiveness of mHealth apps as other medical devices [1].

mHealth offers a unique opportunity to tailor and customize care for individual patients on the basis of health needs and behavioral attributes. Self-monitoring tools and apps are growing faster than more traditional telemedicine interventions because of the ubiquity of smartphones and the minimal development cost of health-related apps in providing flexible, scalable, mobile, and interoperable platforms [2].

### mHealth Patient Engagement

mHealth also promises greater patient engagement, given the technology’s near instant and always-on functionality, and continual use for multiple tasks. This concept is critical to the behavior change required for improved patient outcomes. However, little is known about the health needs or health behaviors targeted by current mHealth offerings. Therefore, in this study, we set out to describe the most popular mHealth apps, both in purpose and engagement method, available in the Apple iTunes marketplace.

## Methods

### Health and Wellness Applications

We sampled a population of health apps that were well characterized with respect to intended use and download statistics using the iTunes Store (Apple Inc). Apple screens apps submitted for distribution through the store for objectionable content and categorizes them based on functionality and developers’ descriptions [3]. We analyzed apps in the “health and wellness” iTunes Store category, which Apple further grouped on the basis of popularity and other attributes.

We first reviewed each app for potential inclusion in the study. We required each app to have an English-language description, to offer its services and functionality in the United States, and to address a health factor or condition that was gender-neutral with regard to the user. We sought to identify 100 apps in each of the following 4 subcategories designated in the iTunes Store: (1) most popular free apps, (2) most popular paid apps, (3) most recently added free apps, and (4) most recently added paid apps. We obtained the sample of apps on March 21, 2012.

For each app that met the inclusion criteria, we reviewed the description in the iTunes Store to identify the price, the health factor or condition, and the method of user engagement. For paid apps, we identified the initial price, but did not consider the costs of optional or required paid services offered within the app. Table 1 lists the health factors and their corresponding definitions. A single app could have more than one health factor.

**Table 1:** Health factors and primary engagement methods of reviewed apps.

Health factor	Engagement method^a^				
	Changing personal environment	Goal setting	Reinforcement tracking	Self- monitoring	Other
Fitness/training	0	0	2	172	0
Health resource	0	0	0	27	31
Diet/caloric intake	0	5	0	52	0
Health education	0	3	1	30	2
Stress management	23	0	0	4	0
Sleep	6	0	0	9	0
Mental health	0	0	0	8	0
Smoking cessation	1	0	0	2	1
Pain management	0	0	0	1	1
All factors	30	8	3	305	35

^a^No reviewed apps were categorized into the engagement methods of facilitating social support, progress tracking, social presentation or announcement, and social referencing.

### Categorizing the Applications

We also categorized the apps with respect to the type of behavioral strategy or method of user engagement employed. We defined user engagement as the psychological framework used by the app to promote the desired health outcome. We derived unique categories of user engagement based on the principles of the transtheoretical model of change and traditional behavior modification models that consider both individual and social influences [4-11]. We characterized each app according to its primary engagement approach and, in some cases, a secondary engagement approach. The 9 categories of engagement were as follows: (1) changing personal environment, the app modifies the environment to encourage the desired behavior (eg, white or ambient noise, soothing sounds, or images for meditation); (2) facilitating social support, the app creates a group or uses existing groups online or in person and stresses camaraderie, problem solving, solidarity, etc; (3) goal setting, the app facilitates goal setting (eg, weight loss target, fitness goal); (4) progress tracking, the user identifies a goal and the app creates subsidiary goals or tasks based on the user-defined goal and logs the user’s progress; (5) reinforcement tracking, the app allows a third party to assign reinforcements based on information collected by the app regarding the user’s health or health behaviors; (6) self-monitoring, the user tracks his or her behavior in the app with no explicit reference to a goal, the app is simply a tracking tool (eg, pedometer); (7) social presentation or announcement, the app provides implicit social reinforcement, for example, by announcing an action, achievement, or process via social media or an app tool; (8) social referencing, the app facilitates indexing of the user’s behavior in comparison with others, such as in an online community or social group that uses the same tool; and (9) other, the app does not fit the other categories.

All categorizations were made on the basis of the description of features and functionality in the app description in the iTunes Store. We used descriptive statistics (frequencies, means, and SDs) to describe characteristics of the apps according to price, health factors, and behavioral engagement.

## Results

### Reviewed Applications

**Figure 1:**
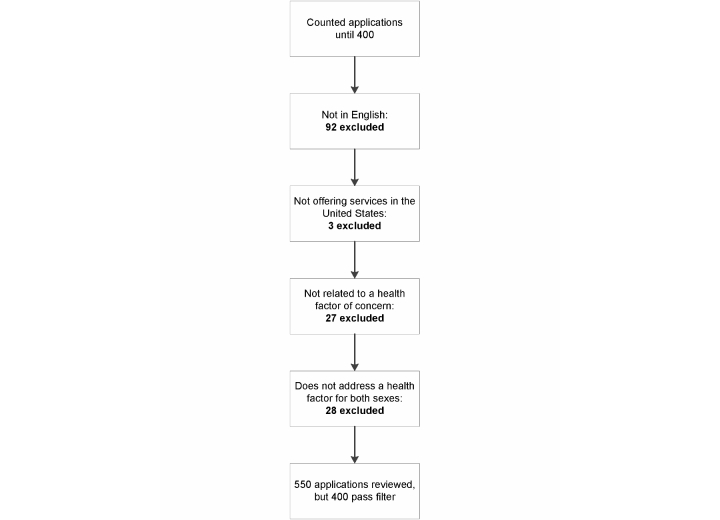
Assessment flowchart.

We reviewed 550 apps, of which 150 did not meet the inclusion criteria. Figure 1 shows the number of apps excluded at each stage of review. The mean cost of the most frequently downloaded paid apps was US $2.24 (SD $1.30); the mean cost of the most recently available paid apps was US $2.27 (SD $1.60).

**Figure 2:**
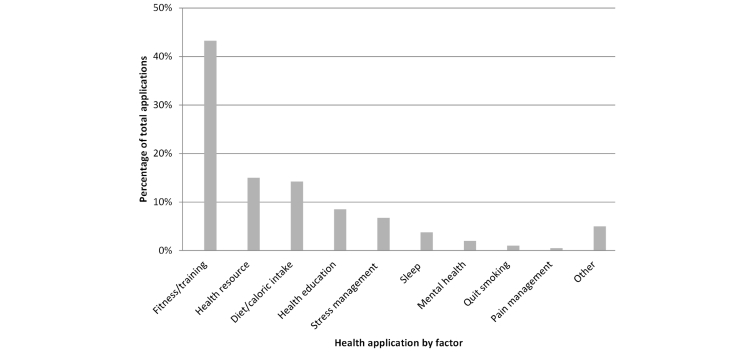
Frequency of health factors in reviewed applications. Note: Fitness/training applications are intended to help improve physical fitness, train for an event, or provide workout/gym plans. Health resource applications locate health resources like doctors, fitness centers, and wait times at medical providers. Diet/caloric intake applications help track calories, assist with making better dietary choices, and help with meal planning. Health education applications provide information about health conditions and wellness topics. Stress management applications inform users about ways to manage stress. Sleep applications inform users on ways to improve sleep patterns. Mental health applications deal with other mental health issues like depression and anxiety. Quit smoking applications inform users about smoking cessation strategies. Pain management applications inform users about pain management strategies.

Figure 2 shows the number of apps that addressed each health factor. Among the 400 apps in the final cohort, fitness/training was the most popular health factor, accounting for 174 out of 400 apps surveyed (43.5%). These apps consisted mostly of predesigned exercise plans or a collection of exercises for the user to follow. The next two most common categories were health resource (ie, providing information about a health resource such as a gym, doctor’s office, or health plan) and diet/caloric intake, accounting for 60 out of 400 apps (15.0%), and 57 out of 400 (14.3%) apps surveyed, respectively. The diet/caloric intake apps most commonly allowed users to track calories consumed and expended. All of the calorie apps had a preexisting database of calorie information, but allowed users to create their own items.

### Engagement Approaches

Table 1 categorizes the reviewed apps by their primary engagement approaches. Self-monitoring was the most common method of engagement approach, as it was utilized in 299 apps out of 400 surveyed (74.8%). A total of 83 apps out of 400 (20.8%) used two or more approaches, with self-monitoring and progress tracking being the most frequent combination.

## Discussion

### Principal Findings

The results of our study provide a baseline for charting the evolution of mHealth apps in prevention and in disease management. Our review of the most frequently used health apps in the iTunes Store highlighted several trends that provide insights into the evolution of mHealth and where gaps and opportunities to improve health status of end users exist. The apps for monitoring exercise regimens and caloric intake (226 apps out of 400 surveyed) dominated the mHealth arena relative to apps addressing chronic issues such as sleep, mental health, and smoking (27 apps out of 400 surveyed). The health apps that deployed self-monitoring apps beyond fitness/exercising training were limited.

Although there is an extensive literature on approaches to behavior change [6-11], and tremendous interest in frameworks offered by the field of behavioral economics [12,13], we found only two major methods of engagement in our sample. The findings echo other recent publications (eg, Cowan et al, Azar et al) [14,15] in which mobile app developers minimally incorporate decades of health behavior theory into app design. These findings reinforce the grassroots entrepreneurial nature of the market and a general lack of awareness of the literature among developers [14-16]. The findings may also reflect the limited insight of consumers into desirable and effective app features. Other possibilities include that the two major methods of engagement are the easiest methods from a programming standpoint and are the methods more likely to be downloaded, acting as a reinforcement mechanism for app developers. Finally, it is unknown whether mHealth approaches to behavior change improve self-monitoring, engagement, or have greater influence on outcomes than traditional models of intervention. The lack of data from these apps that can be analyzed for scientific outcomes further limits opportunities to guide developers in refining their offerings for maximal impact and increased market share.

Although the app market depends on national scale for its financial model, efforts to develop and test apps for specific health conditions and health behaviors may be another path toward popularizing effective apps. To improve customization according to patients’ behaviors, several approaches can be considered including workshops to introduce and explain health behavior methodologies to app developers, grant programs tied to commercialization of behavioral approaches in mHealth, and efforts to assess the impact of mHealth in clinical practice.

### Limitations

Our study has some limitations. We reviewed wellness apps as a potential model for future apps directed at disease management as the market progresses, although these later apps may have more sophisticated approaches to user engagement, and may not be as limited as the wellness apps we reviewed. We did not separately review apps in the Android marketplace due to common apps within the iTunes market, fragmentation in how software is delivered on the platform, and the lack of similar indexing approaches in the Android market. Also, we limited our review to the most frequently downloaded apps. The less popular apps may address different health needs and use different approaches to behavior modification; however, this fact reinforces the notion that apps with scientific foundations may have difficulty differentiating themselves in a crowded marketplace of similar offerings. The market is dynamic and continuously evolving, and our assessment could be quickly outdated by new developments.

### Conclusions

In conclusion, we found that the most popular mHealth apps focused on fitness and self-monitoring. The approaches to patient engagement were limited, presenting an opportunity to improve the effectiveness of the technology. Investments in scientific and developer communities together, and incentives to draw users into specific apps, could alter the dynamics of the market and have a significant impact on health outcomes. Further work will be required to develop this technology to the point where it is most likely to have an impact on patient outcomes.

## Abbreviations

app: application
